# miRNA Signature and Dicer Requirement during Human Endometrial Stromal Decidualization *In Vitro*

**DOI:** 10.1371/journal.pone.0041080

**Published:** 2012-07-20

**Authors:** Carlos Estella, Isabel Herrer, Juan Manuel Moreno-Moya, Alicia Quiñonero, Sebastián Martínez, Antonio Pellicer, Carlos Simón

**Affiliations:** 1 Fundación Instituto Valenciano de Infertilidad (FIVI), Valencia University, Instituto Universitario IVI/INCLIVA, Valencia, Spain; 2 Departamento de Biología Molecular, Centro de Biología Molecular ‘Severo Ochoa’, Consejo Superior de Investigaciones Científicas-Universidad Autónoma de Madrid (CSIC-UAM), Madrid, Spain; Michigan State University, United States of America

## Abstract

Decidualization is a morphological and biochemical transformation of endometrial stromal fibroblast into differentiated decidual cells, which is critical for embryo implantation and pregnancy establishment. The complex regulatory networks have been elucidated at both the transcriptome and the proteome levels, however very little is known about the post-transcriptional regulation of this process. miRNAs regulate multiple physiological pathways and their de-regulation is associated with human disorders including gynaecological conditions such as endometriosis and preeclampsia. In this study we profile the miRNAs expression throughout human endometrial stromal (hESCs) decidualization and analyze the requirement of the miRNA biogenesis enzyme Dicer during this process. A total of 26 miRNAs were upregulated and 17 miRNAs downregulated in decidualized hESCs compared to non-decidualized hESCs. Three miRNAs families, miR-181, miR-183 and miR-200, are down-regulated during the decidualization process. Using miRNAs target prediction algorithms we have identified the potential targets and pathways regulated by these miRNAs. The knockdown of Dicer has a minor effect on hESCs during *in vitro* decidualization. We have analyzed a battery of decidualization markers such as cell morphology, Prolactin, IGFBP-1, MPIF-1 and TIMP-3 secretion as well as HOXA10, COX2, SP1, C/EBPß and FOXO1 expression in decidualized hESCs with decreased Dicer function. We found decreased levels of HOXA10 and altered intracellular organization of actin filaments in Dicer knockdown decidualized hESCs compared to control. Our results provide the miRNA signature of hESC during the decidualization process *in vitro*. We also provide the first functional characterization of Dicer during human endometrial decidualization although surprisingly we found that Dicer plays a minor role regulating this process suggesting that alternative biogenesis miRNAs pathways must be involved in human endometrial decidualization.

## Introduction

Implantation of the human embryo in the maternal endometrium is a key step for the successful establishment of pregnancy and requires a dialog between the competent embryo and the receptive endometrium [1]. Stromal decidualization is a critical endometrial process that allows correct trophoblast invasion and placenta formation [2]. In humans, this process is initiated independently of pregnancy, and includes morphological and biochemical changes of the fibroblast-like stromal cells by the action of ovarian steroids 17β-estradiol (E_2_) and progesterone (P_4_). These changes involve extracellular matrix, cytoskeleton and vascular remodeling, secretory transformation of glands, influx of specialized immune cells and differentiation of the endometrial stromal cells into decidual cells [2]. A defective decidualization response is associated with reproductive disorders such as preeclampsia and may cause pregnancy failures [3]. A network of signaling molecules and transcription factors, which controls the decidualization process, has been identified [4], [5], [6] **.** Proteomics and secretomics have been used to model the human decidual interactome [7]. However, very little information is available about the post-transcriptional regulation of this process.

MicroRNAs (miRNAs) are small (22-nt) endogenous non coding double-stranded RNAs that are key players in the post-transcriptional regulation of physiological processes [8]. miRNAs bind to the complementary target sequences in the 3′ untranslated regions (3′UTR) of mRNAs, and direct the translational repression or degradation of target mRNAs [9]. Dicer is an evolutionarily conserved ribonuclease III required for miRNA processing and the synthesis of small interfering RNAs from long double-stranded RNA [10]. The impact of miRNAs on development has been studied using the tissue-specific inactivation of Dicer, and suggests that Dicer is essential for either limb and lung morphogenesis or the development of the female reproductive system [11], [12], [13], [14]. A conditional disruption of Dicer in uteri is unable to support pregnancy in mice after the embryo transfer without affecting estrogen responsiveness or stimulus-induced decidualization [11], [12], [15]. In humans, miRNAs expression profiles have been described for several gynecological conditions, including endometriosis [16], [17] and preeclampsia [18], [19]. However, the function of Dicer and a comprehensive human miRNAs profile in human stromal decidualization have not yet been described.

The aim of this study was to profile the hESC miRNAs expression throughout this process and to analyze the requirements of miRNAs and Dicer during endometrial decidualization. We describe the differential miRNA expression profile during the decidual transformation of the hESC and globally decreased miRNAs function by reducing Dicer levels, and we have examined several defined decidual responses *in vitro*.

## Materials and Methods

### Ethics Statement

This study has been approved by the Institutional Review Board and Ethics Committee of Instituto Universitario-Instituto Valenciano de Infertilidad (Universidad de Valencia, Spain) (0901-EN-023-FD code). Informed written consent has been obtained from each patient prior to tissue collection.

### Endometrial Stromal Cell Isolation. Culture and *in vitro* Decidualization

Endometrial samples were collected on the day of oocyte retrieval from healthy ovum donors aged 19–35 years old (n = 50). Human endometrial stromal cells (hESCs) were isolated as previously described [20]. hESCs were cultured in DMEM/F12 medium containing 10% charcoal stripped fetal bovine serum (FBS) and 0.1% antibiotics. hESCs obtained from 20 different biopsies were plated in 6-well plates. After confluence, the medium was replaced with fresh media containing 2% FBS, supplemented with 5 µg/ml ascorbic acid and 10 µg/ml transferrin.

*In vitro* decidualization was induced by adding progesterone (1 µM) and 17 ß-estradiol (30 nM) for 9 days and the media were renewed every 3 days (n = 20). Control hESCs were cultured in parallel over 9 days without hormonal treatment. Cells and media were collected on day 9.

Confluent hESCs monolayers (n = 20) were also decidualized with 0.5 mM 8-bromo-cAMP (cAMP; Sigma) and 1 µM Medroxy-Progesterone Acetate (MPA, Sigma) for 5 days which achieves a faster differentiated phenotype.

Both decidualization treatments, hormonal (E_2_+P_4_) and cAMP+MPA, promote the characteristic decidualization phenotypes confirmed morphologically and by prolactin (PRL) levels.

### miRNA PCR Array

Of the 20 hESCs decidualized with progesterone and 17 ß-estradiol, we selected three with similar PRL levels on day 9 (138 ng/ml±19.49 compared to 2.38 ng/ml±0.9 in the control) for miRNA expression analysis. Total RNA was extracted using the miRNeasy Mini Kit (Qiagen). The purity and integrity of the total RNA sample were verified with an Agilent Bioanalyzer (Agilent Technologies, Santa Clara, CA). 1 µg of total RNA was reverse transcribed with the RT^2^ miRNA First Strand kit (SABiosciences) and used for each Human Whole Genome miRNA PCR array (SABiosciences). The expression of 704 human miRNAs (Sanger mirBASE Release 14) was tested in 96-well plates in a LightCycler 480 (Roche) and the analysis was done using the manufacture’s integrated web-based software package for the PCR Array System and by employing the ΔΔCt-based fold-change calculations. miRNA 200 b was not included in the 704 miRNAs of the PCR array; thus, we analyzed its expression in the same samples using specific miRNA-200 b primers (Qiagen). Data are the mean of three independent experiments. A Student’s t-test was used to confirm the significance of the results.

### miRNA Mimic Transfection and *in vitro* Decidualization

To overexpress miR-96 and miR-135 b we used the corresponding miRNA mimics and control sequences from SABiosciences. Primary hESCs were plated at a density of 50% and transfected with the corresponding miRNA mimic (50 nM) or the non silencing siRNA (50 nM) with Lipofectamine 2000 (Invitrogen). Cells were incubated with the miRNAs mimics for 24 hrs at 37°C, then cells were subjected to 0.5 mM 8-bromo-cAMP and 1 µM MPA decidual treatment for 48 h, which correspond to 72 h after miRNA mimic treatment. The efficiency of miRNA overexpression (more than 100 fold change compared to control, data not shown) was confirmed by RT-qPCR using specific miR-96 and miR-135 b primers from SABiosciences. The conditioned culture media (CCM) were collected at 48 h after decidual or vehicle treatment and were analyzed by ELISA for IGFBP-1 (Raybiotech) secretion levels. The CCM total protein concentration was measured by the Bradford assay and was used to normalize the IGFBP-1 values.

### Dicer RNA Interference and *in vitro* Decidualization

To silence Dicer, we used Dicer 1 siRNA (SI00300006) and the AllStars Negative Control siRNA as a negative control. All the sequences were obtained from Qiagen. Primary hESCs were plated at a density of 50%. siRNA for Dicer (50 nM) or the non silencing siRNA (50 nM) were mixed with Lipofectamine 2000 (Invitrogen) and DMEM/F12, and were added to the cells. Cells were incubated for 24 hrs at 37°C, then the media were changed to DMEM/F12 containing 2% FBS, 0.1% antibiotics and the decidual stimulus 0.5 mM 8-bromo-cAMP and 1 µM MPA or control vehícule. The decidual treatment was maintained for 48 h and 72 h, which respectively correspond to 72 h and 96 h after Dicer siRNA treatment. Then cells and conditioned culture media (CCM) were collected. Cells were used for Western blot analysis and Dicer protein levels were measured at 72 h and 96 h after siRNA transfection to confirm Dicer silencing.

The conditioned culture media (CCM) were analyzed by ELISA for IGFBP-1 (Raybiotech), PRL (Abnova) and MPIF-1 (RayBiotech) protein levels. The CCM total protein concentration was measured by the Bradford assay and was used to normalize the PRL, IGFBP1 and MPIF values.

### RNA Isolation and Quantitative Real-time PCR

Total RNA was extracted from hESCs using TRIzol reagent (Invitrogen) following the manufacturer’s protocol. Firstly, 1 µg was reverse-transcribed with the M-MLV RT system (Promega) to generate cDNA for the quantitative analyses. Quantitative real-time PCR was performed in triplicate using LightCycler FastStart DNA Master SYBR green I (Roche) in a LightCycler 480. The mRNA level was normalized to GAPDH as a housekeeping gene. The primers sequences used were:

GAPDH, 5′-GAAGGTGAAGGTCGGAGTC-3′and 5′-GAAGATGGTGATGGGATTTC-3′.

DICER, 5′-TGCTATGTCGCCTTGAATGTT-3′ and 5′- AATTTCTCGATAGGGGTGGTCTA-3′ FOXO1, 5′-GCCATGTAAGTCCCATCAGGA-3′and 5′-ATCGGAACAAGAACGTGGAATC-3′ HOXA10, 5′-GAGAGCAGCAAAGCCTCGC-3′ and 5′-CCAGTGTCTGGTGCTTCGTG-3′.

For miRNAs analysis, total RNA was extracted using the miRNeasy Mini Kit and 1 µg of total RNA was reverse-transcribed with the RT^2^ miRNA First Strand kit. Real-time PCR was conducted with the RT^2^ SYBR Green qPCR Master Mix and miRNA levels were normalized to SNORD44 using the ΔΔCt-based fold-change method. The primers for the individual miRNAs and SNORD44 were obtained from SABiosciences.

**Table 1:** miRNAs differentially expressed identified in the *in vitro* decidualization model.

miRNAsup	p value	Foldchange	miRNAs down	p value	Fold change
miR-95	0,001	22,7	miR-146a	0,047	−6
miR-888	0,005	4,1	miR-155	0,02	−3,3
miR-936	0,016	3,9	**miR-181b**	0,005	−3,1
miR-1185	0,016	3,7	miR-181a*	0,021	−3
miR-518f*	0,028	3,6	miR-135b	0,03	−3
miR-548k	0,044	3,3	**miR-181d**	0,004	−2,9
miR-593	0,001	3,2	miR-200c	0,008	−2,9
miR-486-5p	0,046	3,1	miR-141	0,039	−2,8
miR-29c*	0,014	3	miR-182	0,011	−2,7
miR-449b*	0,043	2,9	miR-429	0,019	−2,7
miR-300	0,003	2,8	miR-483-3p	0,049	−2,5
miR-371-5p	0,007	2,8	miR-200a	0,012	−2,5
miR-1224-3p	0,034	2,6	miR-96	0,019	−2,5
miR-891a	0,016	2,5	miR-183	0,012	−2,4
miR-365	0,038	2,5	miR-9	0,028	−2,3
miR-541*	0,044	2,4	miR-30a	0,006	−2,3
miR-409-5p	0,009	2,3	miR-126	0,004	−2,2
miR-33b*	0,01	2,3			
miR-154*	0,034	2,2			
miR-376a*	0,027	2,2			
miR-133a	0,019	2,1			
miR-218-2*	0,043	2,1			
miR-22*	0,041	2,1			
miR-614	0,023	2,1			
miR-369-3p	0,032	2			
miR-185	0,019	2			

List of the 26 more up-regulated miRNAs and the 17 more down-regulated miRNAs identified in the *in vitro* decidualization model. The p values and fold changes are indicated. The miRNAs previously identified by Qian et al, 2009, are shown in boldface.

**Figure 1:**
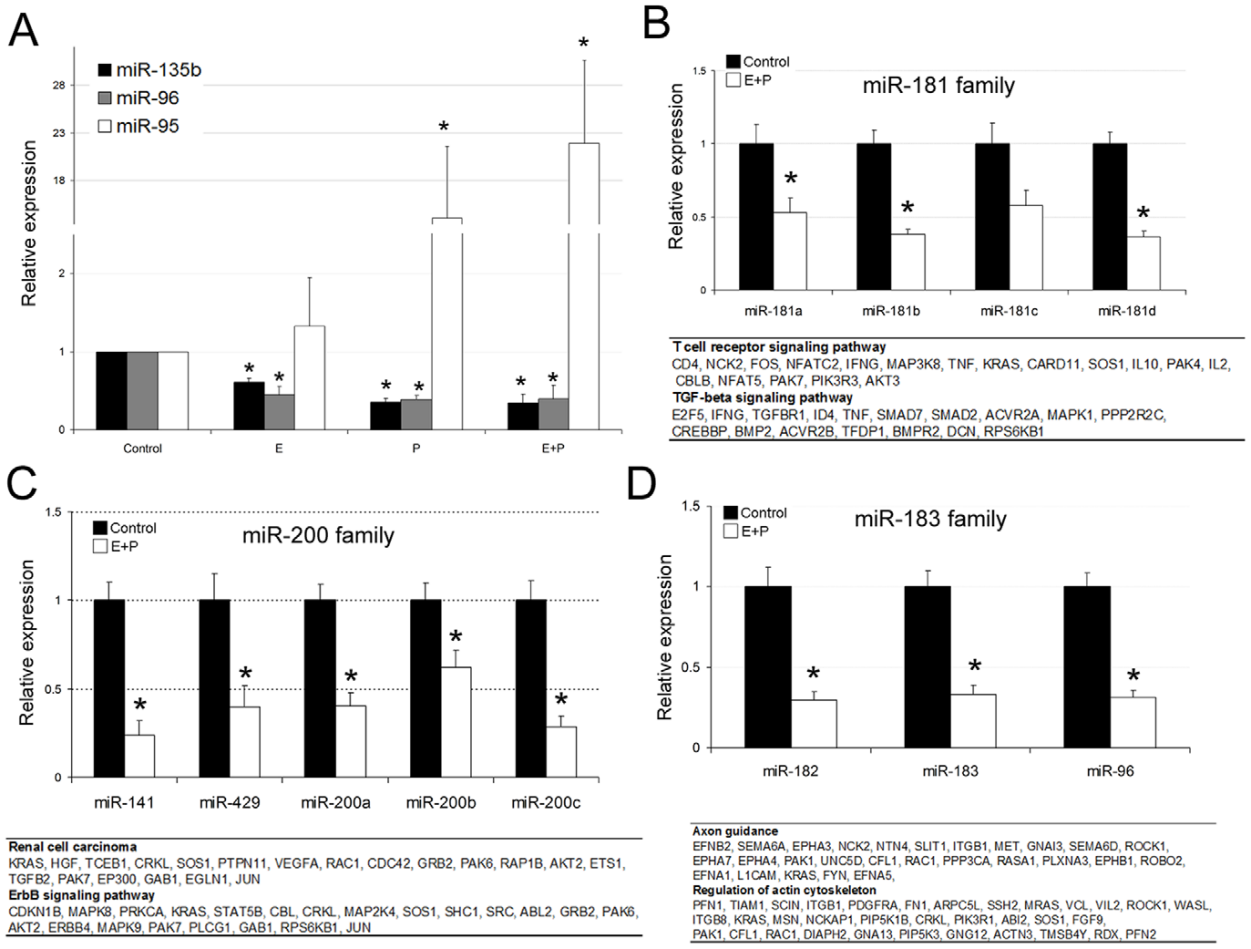
miR-181, miR-183 and miR-200 miRNAs families members are similarly regulated during in vitro decidualization. **A**, miR-95, miR-135b and miR-96 expression levels by quantitative PCR in hESCs treated with 17β-estradiol (E), progesterone (P), or both (E+P), for 9 days. **B**, miR-181 **C**, miR-200 and **D**, miR-183 family members’ expression in the E+P decidualized hESCs for 9 days if compared to the non decidualized control hESCs. **A**, **B**, **C**, and **D**, Data represent the mean of three independent experiments. Error bars represent the standard error of the mean (SEM). Statistical analysis,* p<0.05. The molecular pathways potentially regulated by the mir-181, miR-200 and miR-183 families with the potential target genes are listed below the histograms.

### Western Blots

hESCs were lysed in ice-cold buffer containing 300 mM NaCl, 20 mM Tris, 10 mM EDTA, 2% (v/v) Triton X-100, pH = 7.3 supplemented with a protease inhibitor cocktail (Roche) and PMSF 10 mM (Sigma). The CCM were obtained after exposure of hESC subjected to either Dicer silencing or control siRNA, and to a decidual stimulus (cAMP+MPA) for 72 h. The CCM proteins were precipitated with acetone at −20°C for at least 3 h and were centrifuged at 6000 g. Pellets were resuspended in lysis buffer and supplemented with a protease inhibitor cocktail.

Protein quantification was performed by the Bradford assay. Next, 20 µg/lane of proteins extracts from the cell lysates and CCM were separated on 10% SDS-polyacrylamide gels, transferred to polyvinylidene difluoride membranes (BIO-RAD) and incubated overnight at 4°C with antibodies specific to human DICER (Cell Signaling, 3363), C/EBPβ (Santa Cruz, sc-56637), COX2 (Abcam, ab15191), HOXA10 (Santa Cruz, sc-17159), FOXO1 (Santa Cruz, sc-11350), SP1 (Santa Cruz, sc-420), TIMP-3 (Abcam, ab39184) and β-ACTIN as housekeeping (Santa Cruz, sc-47778). After washing three times, blots were incubated with diluted horseradish peroxidase-conjugated second antibodies (Santa Cruz Biotechnology, St. Cruz, CA) for 1 h at room temperature. Blots were then washed extensively and developed using enhanced chemiluminescence. Ponceau membrane staining was used for data normalization of TIMP-3 secretion levels. Densiometry analysis of the gels was carried out using the ImageJ software (http://rsbweb.nih.gov/ij/links.html) and each Western blot has been normalized to the housekeeping protein band to correct for differences during sample loading.

### Immunohistochemistry and Immunofluorescence

Immunohistochemistry was performed using an LSAB peroxidase kit (Dako). Ten endometrial biopsies from early secretory (5 of day 16) and late secretory (2 of day 24 and 3 of day 26) natural cycles were formalin-fixed, paraffin-embedded, sectioned and mounted on glass slides. After deparaffinization and rehydration, antigen retrieval was performed on 10 mM sodium citrate (pH 6) buffer at 121°C for 3 min. Endogenous peroxidase was abolished by 2% H_2_O_2_ in PBS for 10 min. Sections were blocked for 30 min at RT with 5% of non-fat milk and incubated with 1/100 diluted rabbit anti-DICER antibody (Santa Cruz, sc-30226) at 4°C overnight in a humidifying chamber. In the negative controls, a non-inmune IgG Rabbit isotype (Dako) was used. The secondary antibody (Labeled Polymer-HRP, anti-rabbit) and the DAB chromogen used for staining development were supplied with the Dako kit.

Counterstaining was carried out with hematoxylin and slides were sealed with mounting medium (eukit, Sigma). Immunostaining intensity was evaluated as absent (0), weak (1), moderate (2) or intense (3) by six independent observers in a blinded fashion.

For immunofluorescence, hESCs were fixed in 4% paraformaldehyde in PBS with 0.1% Triton and 0.1% sodium deoxycholate for 25 minutes. Cells were then blocked with PBS 0.3% Triton, 0.03% sodium azide and 1% BSA for 30 minutes. Cells were incubated with primary antibodies (vimentin, Sigma V6630 and E-cadherin, Abcam Ab1416) in blocking solution buffer overnight at 4°C, followed by the appropriate secondary conjugated antibody (Molecular probes) and phalloidin for F-actin staining (fluka 77418) for 1 hr at room temperature. Cells were mounted in Vectashield medium containing DAPI (Vector Laboratories) and images were taken using a Leica sp2 confocal microscope.

### miRNA Target Prediction

To identify potential miRNA target genes, we used four publicly available target prediction algorithms: targetscan (http://www.targetscan.org/), miRanda (microRNA.org), microCosm (http://www.ebi.ac.uk/enright-srv/microcosm/htdocs/targets/v5/) and PicTar (http://pictar.mdc-berlin.de/). The Diana miR-Path database is a web-based computational tool developed to identify the molecular pathways potentially altered by the expression of single or multiple microRNAs [21] (http://diana.cslab.ece.ntua.gr/pathways/).

### Statistics

Data are expressed as means +/− standard error of the mean (SEM). Significance was tested by Student’s *t* test*s* or one-way ANOVA with Dunnett’s post-hoc for comparison of each treated group to the non-treated control when appropriate.

## Results

### miRNA Signature in hESC during *in vitro* Decidualization

Twenty endometrial biopsies were processed and the isolated hESCs were incubated with E_2_ and P_4_ for 9 days. The decidual phenotype was confirmed by the characteristic polygonal cell morphology and the secretion of the decidual marker prolactin (PRL) (Figure S1B and C). We selected three paired control and decidualized hESCs showing an equivalent increase of PRL levels to reduce interindividual variability (Figure S1C). The expression of 704 human miRNAs during endometrial decidualization was assayed by an unbiased genome-wide screen of miRNAs using the miRNA PCR array. We identified the 20 most expressed miRNAs in control and decidualized hESC (Table S1). Some of these highly expressed miRNAs have been previously described in a study by Qian et al, 2009 (10 in our 20 most expressed miRNAs) [22].

Next, we addressed the differential miRNAs expression in hESCs during the decidualization process. The significance analysis of our miRNA PCR array results reveals that a total of 26 miRNAs were up-regulated (fold change >2, p<0.05) and that 17 miRNAs were down-regulated (fold change <-2, p<0.05) in the decidualized hESCs if compared to the non decidualized hESCs (Table 1). Next, we examined the effect of individual ovarian steroids on the regulation of miR-95, this being the most up-regulated miRNA in our *in vitro* decidualization assay, as well as miR-135 b and miR-96. The results indicate that miR-95 was up-regulated by P_4_ and reached maximal levels with E_2_+P_4_, while miR-96 and miR-135b were both down-regulated by E_2_ or P_4_ (Figure 1A).

We used a conservative approach to identify the potential target genes for the differentially expressed miRNAs. This approach is based on the use of four publicly available miRNA target prediction algorithms (targetscan, miRanda, microCosm and PicTar). Only those targets shared by at least two of the four prediction programs were selected and a subset of them has been associated with endometrial stromal decidualization, including transcription factors, growth factors, cytokines and extracellular matrix (ECM) remodelling enzymes [2], [5], [6], [7], [23], [24], [25] (Table 2). We identified potential miRNAs targets involved in differentiation and decidualization events, such as transcription factors (FOXO1, C/EBPß, SP1, HOXA10 or STAT5), interleukins (IL-1A, IL-1B, IL-5) and growth factors and their receptors (VEGFA, EGFR, TGFB-1) (Table 2).

**Table 2:** List of the potential miRNA targets involved during differentiation and decidualization events.

	Target	miRNA Up	miRNA Down
***Transcription factors***	**FOXO1**	miR-486-5p; miR-369-3p	miR-183; miR-135b; miR-96
	**Hoxa10**		miR-135b; miR-182; miR-96
	**Sp1**	miR-369-3p; miR-154*; miR-593;miR-936	miR-135b; miR-155
	**Sp3**	miR-548k; miR-154*; miR-133a;miR-369-3p	miR-141; miR-135b; miR-182; miR-96; miR-30a; miR-155
	**STAT3**	miR-548k	
	**STAT5A**		miR-141; miR-200a; miR-200c
	**STAT5B**		miR-141; miR-200a
	**ETS1**	miR-1224-3p; miR-95	miR-181b; miR-155; miR-9; miR-429; miR-181d; miR-200c
	**CREB1**		miR-96; miR-181b; miR-182; miR-181d
	**CREB3**		miR-181d; miR-181b
	**CREB5**	miR-365; miR-133a	miR-429; miR-200c
	**CEBPB**	miR-369-3p	miR-155
***Growth Factors***	**IGF-1**	hsa-miR-936	miR-483-3p
	**IGF-1R**		miR-182; miR-96
	**IGF-2**		miR-181d
	**IGF-2R**	miR-185	miR-30a; miR-200c
	**VEGFA**		miR-126; miR-429; miR-200c
	**HB-EGF**		miR-96; miR-183; miR-135b; miR-182
	**EGF-R**	miR-133a	miR-141; miR-200a
	**TGFB-1**	miR-593; miR-548k	miR-9; miR-181d; miR-429; miR-181b; miR-200c
	**TGFBR-1**	miR-548k; miR-369-3p; miR-133a	miR-181d; miR-181b
	**TGFB-2**		miR-141; miR-200a
	**TGFB-2R**		miR-155; miR-9
	**LEFTY2**		miR-181d; miR-181b
	**INHBA**		miR-135b
	**LIF**		miR-181b; miR-181d
***Interleukin*** **s**	**IL-1A**		miR-30a; miR-181b; miR-181d; miR-141; miR-200a
	**IL-1B**	miR-888	
	**IL-2**	miR-154*	miR-181b; miR-181d
	**IL-3**		miR-146a
	**IL-5**		miR-429; miR-200c
	**IL-6**	miR-365; miR-371-5p; miR-369-3p	
	**IL-7**	miR-376a*	miR-181d
	**IL-8**		miR-182
	**IL-10**	miR-888	
	**IL-11**	miR-154*	
	**IL-12A**		miR-9
	**IL-12B**		miR-183
	**IL-13**	miR-300	miR-155
***ECM remodelling enzymes***	**LAMC1**	miR-95; miR-548k	miR-96; miR-182; miR-429; miR-200c; miR-181d; miR-181b
	**TIMP-2**	miR-891a; miR-369-3p	miR-429; miR-200c; miR-30a
	**TIMP-3**		miR-181b; miR-181d; miR-30a
	**TIMP-4**		miR-146a; miR-96
	**FN1**		miR-200c; miR-429; miR-96; miR-182

To gain insight into the molecular pathways potentially regulated by the miRNAs identified in our *in vitro* decidualization model, we used the Diana miR-Path database [21]. The top five pathways, which are more represented by the down-regulated miRNAs targets, include axon guidance, adherens junction, actin cytoskeleton regulation, ErbB (EGFR) signaling and renal cell carcinoma. Similarly, the targets of the up-regulated miRNAs group cluster in the top five pathways, including actin cytoskeleton regulation, adherens junction, axon guidance, Wnt signalling and MAPK pathways.

**Figure 2:**
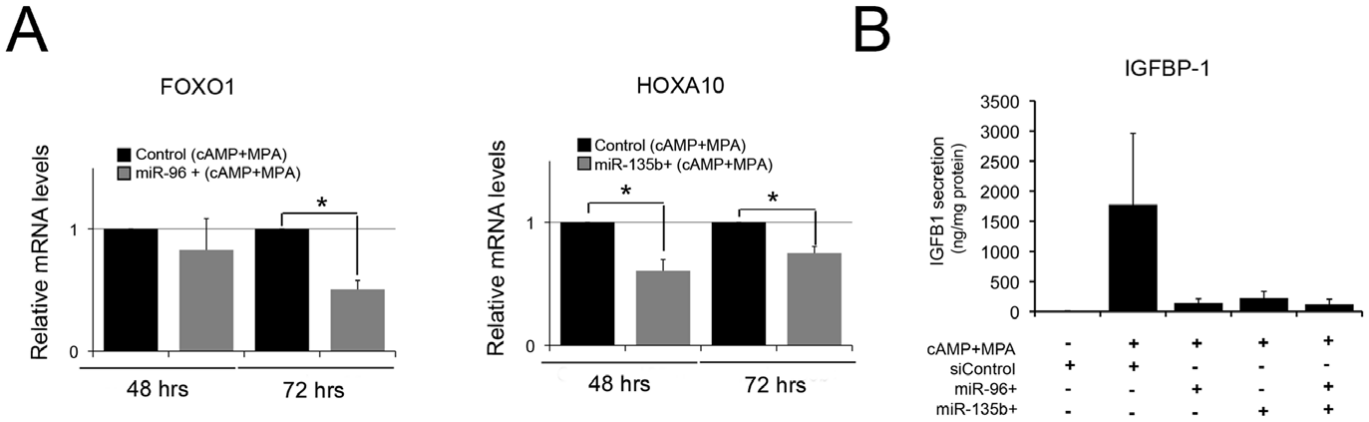
miR-96 and miR-135b role during endometrial decidualization. **A,** Relative FOXO1 and HOXA10 mRNA levels quantified by RT-qPCR in control or miR-96 and miR-135b transfected mimics in decidualized hESCs 48 h and 72 h after decidual treatment. **B,** IGFBP-1 secretion levels in the non decidualized control hESCs, the control miRNA decidualized hESCs and miR-96, miR-135b or miR96 and miR-135b transfected mimics in decidualized hESCs measured by ELISA at 48 h after decidual treatment. IGFBP-1 secretion levels were normalized to the total amount of protein present in the media. Data represent the mean of three independent experiments. Error bars represent the SEM. Statistical analysis, * p<0.05.

### miR-181, miR-183 and miR-200 miRNAs Families Members are Similarly down-regulated during *in vitro* Decidualization

Interestingly, three miRNAs families (miR-181, miR-183 and miR-200) were down-regulated during the decidualization process. For the mir-181 family, which includes six miRNAs precursors (mir-181a-1, mir-181a-2, mir-181b-1, mir-181b-2, mir-181c and mir-181d) located at three different loci (chromosomes 1, 9 and 19), the corresponding mature miRNA expression in decidual cells decreased (Figure 1B). These miRNAs share an identical seed region, suggesting that they regulate similar targets. By using the Diana miR-Path database [21], we searched for the molecular pathways potentially regulated by the mir-181 family. The two main pathways with more potential share targets are the T cell receptor and TGFß signaling pathways (Figure 1B). Another miRNA family that is down-regulated during the decidualization process is the mir-200 family. This family is located at two different loci (chromosomes 1 and 12) and includes the mir-141, mir-429, mir-200a, mir-200b and mir-200c, miRNAs that were significantly down-regulated in the decidualized hESCs (Figure 1C). These miRNAs share an identical seed region except for miR-141 and miR-200a which change one nucleotide at position 4. The number of shared potential targets was low and was included in the cell cycle pathway. We also analyzed the more enriched molecular pathways by the union of miRNAs targets for the 4 mir-200 family members. The most represented pathway was the renal cell carcinoma and ErbB signaling. Three additional miRNAs (miR-96, miR-183 and miR-182), clustered together at the same locus (chromosome 7), were significantly down-regulated during decidualization (Figure 1D). miR-182 and miR-96 share an identical seed, while miR-183 only differ from the others in one nucleotide at position 2. The pathways regulated by the potential targets of these miRNAs are the axon guidance and cytoskeleton regulation. Collectively, our data indicate that decidualized hESCs have a specific miRNA signature if compared to non decidual cells.

### miR-96 and miR-135b Regulates FOXO and HOXA10 Expression and IGFBP-1 Secretion

We have selected miR-96 and miR-135b, two miRNAs that decrease their expression during stromal differentiation (Table 1), to evaluate in more detail their function during endometrial decidualization. It has been previously demonstrated that miR-96 and miR-135b regulate the expression of their target genes, FOXO1 and HOXA10 [26], although their role in this particular process has not been studied. These two proteins have been proposed to interact and cooperatively stimulate IGFBP-1 promoter in primate endometrial cells [26]. Increased levels of miR-96 and miR-135b using miRNAs mimics in hESCs under the decidualization treatment reduced the expression of FOXO1 and HOXA10, respectively and decrease IGFPB-1 secretion levels (Figure 2A and B). Although IGFBP-1 reduction is not statistical significant all the biopsies analyzed (n = 3) show at least a 5 fold decrease after miRNA mimic transfection.

**Figure 3:**
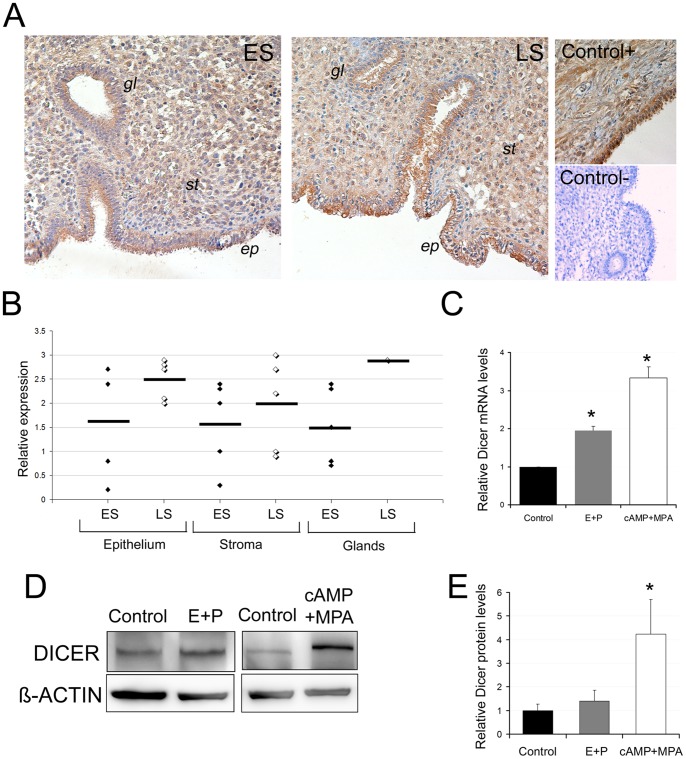
Dicer expression is regulated during endometrial decidualization. **A**, Dicer staining in two representative endometrial biopsies from the early secretory (ES) and late secretory (LS) menstrual cycle phases. An ovarian biopsy was also used as a positive control. For the negative control, we used an endometrial biopsy with a non inmune IgG Rabbit isotype. *ep*, ephithelium, *st*, stroma and *gl*, glands. **B**, Schematic representation of Dicer expression levels in 10 endometrial biopsies (5 ES and 5 LS) in the stroma, luminal epithelia and glands. The relative expression value was the average expression score (from 0 to 3; from low to high) given for each of the endometrial biopsies by six independent observers. **C**, Dicer mRNA levels were up-regulated by the different decidual stimuli: cAMP+MPA for 5 days or 17β-estradiol and progesterone (E+P) for 9 days. **D**) A representative Western blot of DICER and ß-ACTIN in the control and E+P- or cAMP+MPA-treated cells. Note that the DICER band in cAMP+MPA shifts upwardly, probably due to a post-transcriptional modification. **D**) Quantification of DICER protein levels in cAMP+MPA- (5 days) or in E+P- (9 days) treated cells if compared to the control cells. **C** and **D**, Data represent the mean of three independent experiments. Error bars represent the SEM. Statistical analysis,* p<0.05.

### Functional Relevance of Dicer in hESCs during *in vitro* Decidualization

We first assessed the *in vivo* localization of Dicer at the protein level in 10 endometrial samples obtained in two different menstrual cycle phases: non decidualized early secretory (ES) (n = 5) and decidualized late secretory (LS) (n = 5). Dicer levels were readily detectable in the stroma, the luminal epithelium and the glandular compartments (Figure 3A). A semi-quantitative evaluation of Dicer staining was scored blindly by six different observers. The strength and the number of Dicer-positive cells were scored from 0 to 3 (from low to high) in the three different compartments. In general, Dicer staining tended to be higher in all the endometrial compartments during the LS phase if compared to ES (Figure 3B).

Next, we analyzed Dicer mRNA and protein levels in isolated hESCs with different decidualization treatments. We employed two widely accepted inducers of the decidual reaction; ovarian steroids E_2_ and P_4_ and the non hormonal cAMP stimulus [6], [7], [27], [28]. Dicer mRNA levels were significantly up-regulated after any decidualization treatment used (Figure 3C), although the cAMP and MPA stimuli significantly increased Dicer at the protein level (Figure 3D and E). These results suggest that Dicer expression in hESC increases during *in vitro* decidualization.

**Figure 4:**
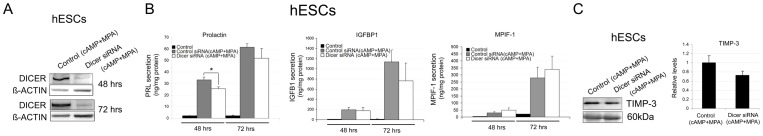
Dicer function during endometrial decidualization. **A**, A representative Western blot of DICER protein levels in control siRNA decidualized hESCs (Control siRNA (cAMP+MPA)) and Dicer knockdown decidualized hESCs (Dicer siRNA (cAMP+MPA)) at 48 h and 72 h after decidual treatment. **B**, Prolactin, IGFBP-1 and MPIF-1 secretion levels in the non decidualized control hESCs, the control siRNA decidualized hESCs (Control siRNA (cAMP+MPA)) and the Dicer knockdown decidualized hESCs (Dicer siRNA (cAMP+MPA)) measured by ELISA at 48 h and 72 h after decidual treatment. Prolactin, IGFBP-1 and MPIF-1 secretion levels were normalized to the total amount of protein present in the media. Data represent the mean of four independent experiments. Error bars represent the SEM. Statistical analysis, * p<0.05. **C**, TIMP-3 secretion levels measured by Western blot in the control siRNA decidualized hESCs (Control siRNA (cAMP+MPA)) and the Dicer knockdown decidualized hESCs (Dicer siRNA (cAMP+MPA)) at 72 h after decidual treatment. Ponceau staining of membranes was used as a protein-loading control. Only the 60 kDa band of the ponceau gel is shown. Data represent the mean of four independent experiments. Error bars represent the SEM.

To demonstrate the functional relevance of endogenous Dicer during *in vitro* decidualization, we developed a siRNA approach to reduce Dicer levels in hESCs subjected to the decidualization stimulus. Transfection of hESCs with Dicer siRNA dramatically decreased Dicer protein levels at 48 h and 72 h after the decidualization treatment (Figure 4A).

To examine the potential functions attributed to miRNAs during *in vitro* decidualization, we measured the levels of the defined decidual secreted markers PRL, IGFBP1, TIMP-3 and the newly identified MPIF-1 [7], [29], [30] at 48 h and 72 h after initiating the decidual treatment in hESCs with decreased Dicer function (Figure 4B and C). The PRL, IGFBP-1, and MPIF-1 secretion levels significantly increased after treatment with cAMP and MPA when compared to the non decidualized hESCs (Figure 4B). Dicer siRNA knockdown did not significantly reduce the PRL, IGFBP-1 or MPIF-1 levels at 48 h and 72 h after the decidual treatment, except for the PRL levels, which slightly but significantly lowered at 48 h (Figure 4B). Similarly, TIMP-3 secretion levels were up-regulated after decidualization (data not shown), but did not significantly lower in the Dicer siRNA knockdown hESCs (Figure 4C).

**Figure 5:**
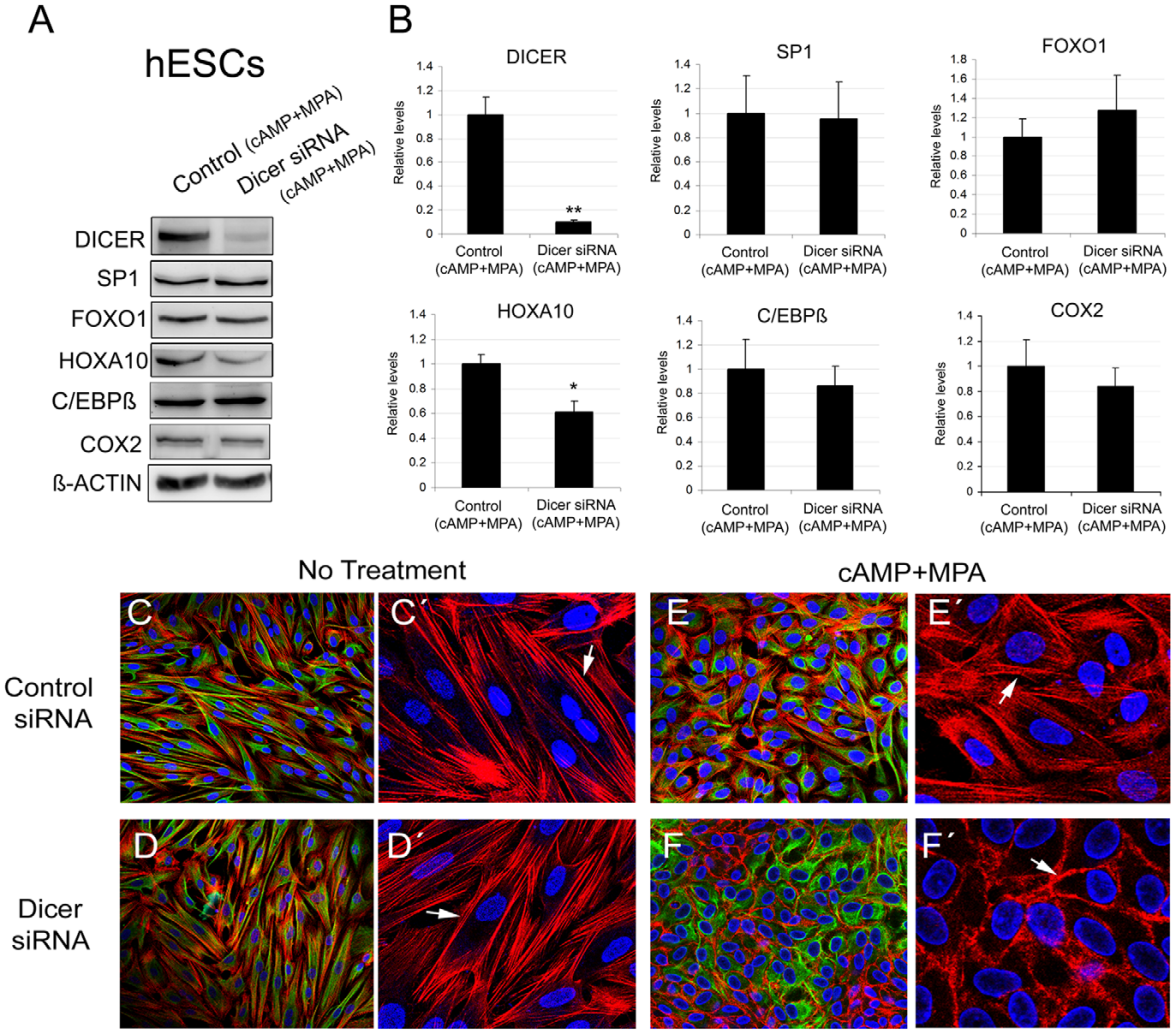
Expression of the transcription factors and enzymes which play a prominent role during decidual transformation in Dicer knockdown hESCs and role of Dicer in the morphological transformation of decidual hESCs. **A**, representative Western blot of DICER, SP1, FOXO1, HOXA10, C/EBPß and COX2 protein levels in the control siRNA decidualized hESCs (Control siRNA (cAMP+MPA)) and the Dicer knockdown decidualized hESCs (Dicer siRNA (cAMP+MPA)) at 72 h after decidual treatment. **B**, Western blot quantification of DICER, SP1, FOXO1, HOXA10, C/EBPß and COX2 protein levels in the control siRNA decidualized hESCs (Control siRNA (cAMP+MPA)) and the Dicer knockdown decidualized hESCs (Dicer siRNA (cAMP+MPA)) at 72 h after decidual treatment. Data represent the mean of four independent experiments. Error bars represent the SEM. Statistical analysis, * p<0.05 and **p<0.01. **C**–**F**, F-actin (Red), Vimentin (Green) and Dapi (Blue). **C**, Control siRNA hESCs without decidual treatment. Note the fibroblast-like shape of the stromal cells and the longitudinal orientation of the actin filaments (arrow). **D**, Dicer siRNA hESCs with no decidual treatment. No apparent morphological change was observed. **E**, Control siRNA hESCs treated with cAMP+MPA for 72 h. Note the characteristic polygonal cell shape of decidual cells and the random orientation of the actin filaments (arrow). **F**, Dicer siRNA hESCS treated with cAMP+MPA for 72 h. Decidual transformation was still apparent while the cells were rounder than the control siRNA decidualized cells. Note the peripheral distribution of the actin filaments (arrow).

Increased cellular cAMP levels sustained the activation of signaling pathways and downstream transcription factors required for the morphological and biochemical differentiation of decidual stromal cells. Therefore, we investigated whether the expression of some of the transcription factors and enzymes that play a prominent role during the decidual transformation, such as FOXO1, C/EBPß, COX2, SP1 and HOXA10 [31], [32], [33] [34], [35], [36], [37], was affected in decidualized hESCs after Dicer knockdown. Western blot analysis of the decidualized Dicer siRNA transfected primary hESCs showed no statistical change in the protein levels of FOXO1, C/EBPß, COX2 and SP1 (Figure 5A and B). In contrast, HOXA10 levels significantly diminished in the decidual Dicer knockdown hESCs (Figure 5A and B).

The transformation of fibroblast-like hESCs into a rounded epithelioid shape is a phenotypic landmark observed during decidualization, both *in vitro* and *in vivo* [30]. We investigated whether Dicer was required for this characteristic morphological transformation. Dicer knockdown in hESCs changed neither the shape nor the longitudinal orientation of the F-actin filaments if compared to the control cells (Figure 5C and D). Decidualized hESCs displayed polygonal cell morphology with a random distribution of F-actin filaments when compared to the non decidualized hESCs (Figure 5C and E). However, the Dicer knockdown in hESCs subjected to the decidual stimulus presented an even rounder phenotype than the decidualized siRNA control cells, and the actin filaments displayed a peripheral distribution (Figure 5F). Altogether, these results suggest that Dicer plays a minor role during *in vitro* decidualization, at least for the decidual markers analyzed.

## Discussion

This study describes the miRNA signature during human endometrial stromal *in vitro* decidualization and analyzes the role of Dicer, a major component of miRNA biogenesis machinery, during this process. A previous study using microarray technology described the expression of 435 human miRNAs in *in vitro* decidualized stromal cells using cAMP and MPA as decidual stimuli [22]. Surprisingly, from the total 33 differentially expressed miRNAs during our *in vitro* decidualization model, only two (miR-181b and miR-181d) have been previously identified in the Qian et al study [22]. The low number of miRNA coincidences between these two studies may be due to the different decidualization stimulus used (E_2_+P_4_ *vs.* cAMP+MPA), the expression profile technique employed (PCR array *vs.* microarray) and the number of miRNAs tested (704 *vs.* 435). Some of the molecular pathways potentially altered by the miRNAs identified in our *in vitro* assay are the axon guidance, adherens junction and actin cytoskeleton regulation. These pathways are highly involved in the regulation of actin filaments, a key process required for the correct differentiation of endometrial stromal cells, including potential target genes such as Rac1, CDC42, RhoA or ROCK1 [38], [39]. Other potentially regulated pathways are the ErbB (EGFR) signaling and the renal carcinoma pathways, which have been implicated in cell differentiation, migration and angiogenesis, including potential targets such as VEGFA and TGFß2, molecular factors that are regulated during endometrial decidualization [4], [6], [40]. Interestingly, the transcription factor HOXA10, which is essential for female fertility and decidualization, at least in mice [41], [42], is a validated target of miR-135b [43], one of the miRNAs which was down-regulated during our *in vitro* decidualization assay. Moreover, our results indicate that HOXA10 is regulated by miR-135b and its levels are significantly lower after Dicer knockdown in decidualized hESCs. These findings support the regulation of HOXA10 by miRNAs. Another interesting finding is that all the members of three different miRNAs families (miR-181, miR-200 and miR-183) have been identified to be similarly regulated. These miRNA families are clustered together in the genome, which provides a possible explanation for their co-regulation and their highly related sequence between the members of each family. Therefore, miRNAs family members are proposed to regulate similar targets and pathways in a coordinated and cooperative manner. The top two molecular pathways potentially regulated by the miR-181 family are TGFß signaling and T cell receptor. The TGFß signaling pathway has been shown to play a role in stromal cells undergoing decidualization [44], [45]. Some of the potential targets include TGFß receptor 1, the downstream effector SMAD2 and the CREB-binding protein (CREBBP), a coactivator of the cAMP-response element-binding protein (CREB) [46], [47]. The T cell receptor pathway has also been reported to be implicated in this process by promoting allograft tolerance and, therefore, pregnancy success [48]. One validated target of miR-181b in mice is TIMP-3, an inhibitor of metalloproteases and a characteristic decidualization marker [49].

The molecular pathways potentially regulated by the miR-200 family include renal cell carcinoma and ErbB signaling. The former includes potential target genes such as VEGFA, implicated in angiogenesis, and genes regulating the actin cytoskeleton such as RAC1. Both of these events are highly involved in the decidualization process [39], [40]. The other most representative pathway is ErbB signaling, which includes STAT5b that is induced and translocated to the nucleus during decidualization, and which binds to the PRL promoter [50]. Moreover, the transcription factor ZEB1, a validated target of the mir-200 family, is up-regulated during the secretory phase in the human endometrial stroma, and its misregulation has been implicated in endometrial cancer progression [51], [52].

The other miRNA family includes miR-96, miR-182 and miR-183 with potential molecular pathways implicated in actin cytoskeleton reorganization, such as RAC1 and ITGß1, and both genes are implicated in endometrial decidualization [39], [53]. Moreover, the decidual factor FOXO1, is negatively regulated by miR-182 and miR-96 [54]. Our results extend these data indicating that in decidualized hESCs, miR-96 regulates FOXO1 expression.

Moreover, the overexpression of miR-96 or miR-135b individually or combine, two downregulated miRNAs during endometrial decidualization, has an impact on IGFBP-1 secretion, suggesting that precise and coordinated miRNA regulation is required for a correct decidualization response.

Aberrant miRNA expression has been associated with very different human diseases, including reproductive conditions such as endometriosis, preeclampsia and endometrial cancer [55]. Noteworthily, some of the differentially expressed miRNAs identified in our study during decidualization have been found to be misregulated in endometriosis (e.g., miR-9, miR-135b and miR-141) [56], [17], [43], preeclampsia (e.g., miR-155, miR-183 and miR-181b) [18], [19], [57] or endometrial cancer (e.g., the miR-200 family and miR-96) [54], [58], [59].

Dicer has proved essential for female fertility [11], [12], [15]. Previous results have suggested that the uterine decidual response is not affected in conditional Dicer knockdown mice, at least for the expected transformation of stromal fibroblasts-like cells into epithelioid-like cells [12]. However, the role of Dicer during human endometrial decidualization has not yet been studied. Our results indicate that Dicer expression is up-regulated during decidualization. To determine the global role of miRNAs during *in vitro* decidualization, we significantly lowered Dicer levels in hESCs. We found that Dicer plays a minor role during *in vitro* decidualization, at least for the decidual markers analyzed at the exception of HOXA10 which levels decreased after Dicer knockdown. Remarkably, HOXA10 mutant mice are infertile and show a diminished decidualization response, while low HOXA10 levels in humans are associated with endometriosis, which is partly due to an up-regulation in miR-135b levels [41], [43], [60].

The lack of a strong phenotype after Dicer silencing might be due the limitations of our *in vitro* decidualization assay and to the limited decidualization markers available. While endometrial stromal cells decidualize *in vivo* approximately 10 days after post-ovulatory exposure to progesterone, the faster *in vitro* decidualization protocol (cAMP and MPA) used in our experiments could bypass some Dicer or miRNA requirements that are not detectable in this study. Also, the endogenous miRNAs’ long half-life [61] and the existence of alternative miRNA biogenesis pathways, which may maintain active miRNAs in Dicer mutants [62] could explained the lack of phenotype after Dicer knockdown. It is also worth noting that different core miRNA components mutants do not always present the same similar phenotypes to those reported for Dicer and Dgcr8 conditional knockout which hints at the existence of alternative mechanisms that generate functional microRNAs [63].

In conclusion, our results provide a more up-to-date specific miRNA signature of hESC during the decidualization process *in vitro*. We also provide the first functional characterization of Dicer during human endometrial decidualization although, surprisingly, we found that Dicer plays a minor role in regulating this process. These results suggest that Dicer alternative biogenesis miRNAs pathways must regulate the decidual transformation of stromal cells. Moreover, our findings provide potential new biomarkers and therapeutic targets for diseases associated with defective endometrial decidualization.

## Supporting Information

Figure S1 **Decidual phenotypes of hESCs. A**, An endometrial biopsy stained with hematoxylin and E-cadherin that marks the epithelia compartment. Biopsies were subjected to mild collagenase digestion to isolate human endometrial stromal cells (hESCs) from human endometrial epithelial cells (hEECs). The purity of the cultures was assessed by vimentin (Red) (hESCs+, hEECs-) and E-cadherin (Green) staining (hESCs-, hEECs+). Nuclei were marked by Dapi staining (Blue). **B**, Decidual transformation of the endometrial stromal cells after treatment with E+P for 9 days. Note the morphology change of the fibroblast-like cells to the characteristic polygonal cell shape of decidual cells. F-actin (Red), Vimentin (Green) and Dapi (Blue). **C**, PRL secretion levels in the non decidualized control hESCs and the decidualized (E+P) hESCs for 9 days. PRL secretion levels were normalized to the total amount of protein present in the media. Data represent the mean of four independent experiments. Error bars represent the SEM. Statistical analysis, ** p<0.01.
(TIF)

Table S1 List of the 20 most expressed miRNAs in the control and decidualized hESCs in relation to housekeeping gene SNORD44 (normalized to 1). The miRNAs previousy identified by Qian et al, 2009, are denoted in boldface.
(TIF)
