# Supplementary figures and images for: miRNA Signature and Dicer Requirement during Human Endometrial Stromal Decidualization In Vitro
Source: PLoS One. 2012 Jul 20;7(7):e41080. doi: 10.1371/journal.pone.0041080 (PMC3401238; doi:10.1371/journal.pone.0041080)

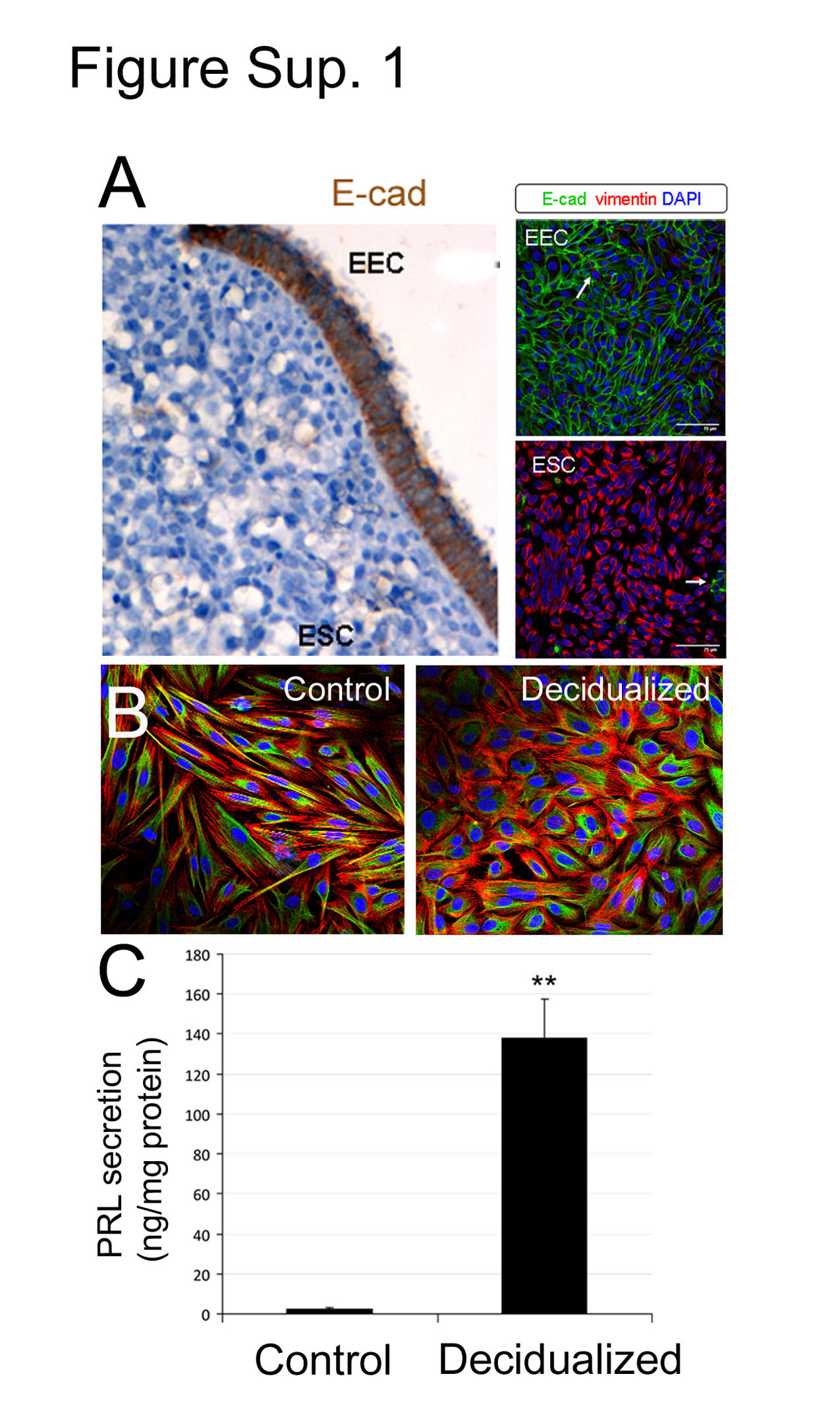

Supplement: Figure S1 — Decidual phenotypes of hESCs. A, An endometrial biopsy stained with hematoxylin and E-cadherin that marks the epithelia compartment. Biopsies were subjected to mild collagenase digestion to isolate human endometrial stromal cells (hESCs) from human endometrial epithelial cells (hEECs). The purity of the cultures was assessed by vimentin (Red) (hESCs+, hEECs-) and E-cadherin (Green) staining (hESCs-, hEECs+). Nuclei were marked by Dapi staining (Blue). B, Decidual transformation of the endometrial stromal cells after treatment with E+P for 9 days. Note the morphology change of the fibroblast-like cells to the characteristic polygonal cell shape of decidual cells. F-actin (Red), Vimentin (Green) and Dapi (Blue). C, PRL secretion levels in the non decidualized control hESCs and the decidualized (E+P) hESCs for 9 days. PRL secretion levels were normalized to the total amount of protein present in the media. Data represent the mean of four independent experiments. Error bars represent the SEM. Statistical analysis, ** p<0.01. (TIF) [file pone.0041080.s001.tif]

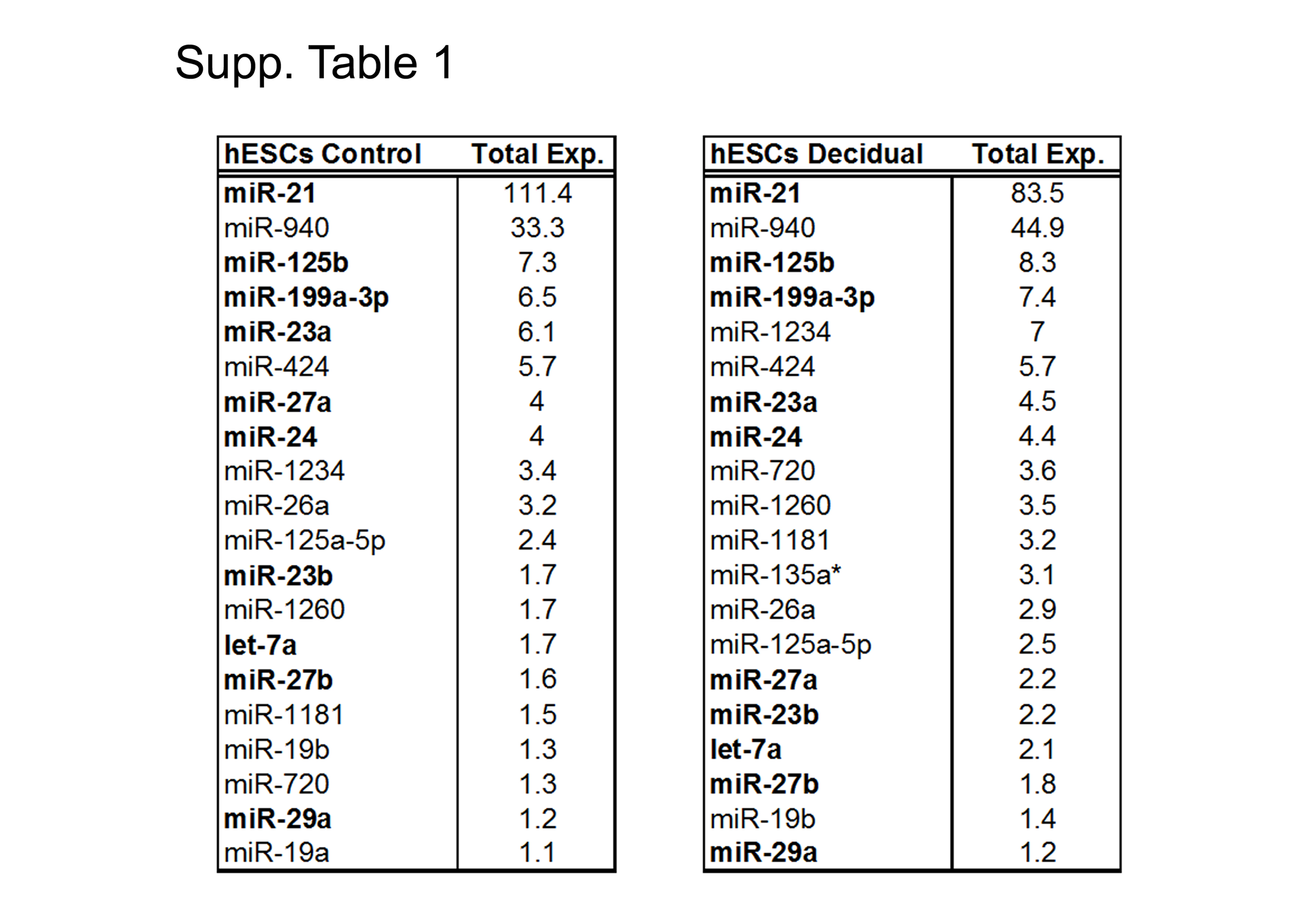

Supplement: Table S1 — List of the 20 most expressed miRNAs in the control and decidualized hESCs in relation to housekeeping gene SNORD44 (normalized to 1). The miRNAs previousy identified by Qian et al, 2009, are denoted in boldface. (TIF) [file pone.0041080.s002.tif]
